# Osteogenesis Enhancement with 3D Printed Gene-Activated Sodium Alginate Scaffolds

**DOI:** 10.3390/gels9040315

**Published:** 2023-04-07

**Authors:** Maria Khvorostina, Anton Mironov, Irina Nedorubova, Tatiana Bukharova, Andrey Vasilyev, Dmitry Goldshtein, Vladimir Komlev, Vladimir Popov

**Affiliations:** 1 Institute of Photon Technologies of Federal Scientific Research Centre “Crystallography and Photonics”, Russian Academy of Sciences, Moscow 108840, Russia; khvorostina.m@gmail.com (M.K.); scftlab@gmail.com (A.M.); 2 Research Centre for Medical Genetics, Moscow 115478, Russia; irina0140@gmail.com (I.N.); bukharova-rmt@yandex.ru (T.B.); vav-stom@yandex.ru (A.V.); dvgoldshtein@gmail.com (D.G.); 3 Central Research Institute of Dental and Maxillofacial Surgery, Moscow 119021, Russia; 4 A.A. Baikov Institute of Metallurgy and Materials Science, Russian Academy of Sciences, Moscow 119334, Russia

**Keywords:** osteogenesis, bone regeneration, sodium alginate, plasmid DNA, gene-activated hydrogel scaffolds, 3D printing

## Abstract

Natural and synthetic hydrogel scaffolds containing bioactive components are increasingly used in solving various tissue engineering problems. The encapsulation of DNA-encoding osteogenic growth factors with transfecting agents (e.g., polyplexes) into such scaffold structures is one of the promising approaches to delivering the corresponding genes to the area of the bone defect to be replaced, providing the prolonged expression of the required proteins. Herein, a comparative assessment of both in vitro and in vivo osteogenic properties of 3D printed sodium alginate (SA) hydrogel scaffolds impregnated with model EGFP and therapeutic BMP-2 plasmids was demonstrated for the first time. The expression levels of mesenchymal stem cell (MSC) osteogenic differentiation markers *Runx2*, *Alpl,* and *Bglap* were evaluated by real-time PCR. Osteogenesis in vivo was studied on a model of a critical-sized cranial defect in Wistar rats using micro-CT and histomorphology. The incorporation of polyplexes comprising pEGFP and pBMP-2 plasmids into the SA solution followed by 3D cryoprinting does not affect their transfecting ability compared to the initial compounds. Histomorphometry and micro-CT analysis 8 weeks after scaffold implantation manifested a significant (up to 46%) increase in new bone volume formation for the SA/pBMP-2 scaffolds compared to the SA/pEGFP ones.

## 1. Introduction

The treatment of critical-sized bone defects resulting from trauma, degenerative diseases, or surgical intervention is still challenging and considered as a serious problem in orthopedics, dentistry, and plastic surgery [1,2,3]. The development of bone graft substitutes to overcome the limitations of currently existing materials and approaches, such as auto- and allotransplantation, is an advanced and exciting area of extensive scientific and clinical research [4,5]. Biomimetic scaffolds, primarily based on biopolymers, such as collagen, hyaluronic acid, and alginates, not only provide a minimal adverse immune response and are available in virtually unlimited quantities, but may also possess desirable osteoinductive and osteoconductive properties for more efficient bone reconstruction [6,7]. The combination of osteoinductive growth factors and osteoconductive hydrogel scaffolds tends to meet the requirements for synthetic bone substitutes in full, providing protein bioactivity in the implantation zone, as well as a microenvironment for newly formed tissue [8,9].

Bone morphogenetic proteins (BMPs) are considered to be crucial biomolecules for stimulating and promoting osteogenic differentiation in cells [10,11]. Though direct protein delivery is considered to be rather effective [12], it can suffer from protein instability in vivo and requires high protein doses to reach a sufficient level of scaffold bioactivity [13]. Gene therapy is one of the most promising alternative approaches in regenerative medicine for bone defect treatment [14]. DNA-encoding osteogenic growth factors in combination with cationic polymers appear to be an effective way of gene delivery, providing safe genome editing and prolonged protein expression [15,16].

To control the duration and location of gene expression, biocompatible and bioresorbable polymer scaffolds (both of natural and synthetic origins) with embedded plasmid DNA are commonly used [17,18,19]. Sodium alginate (SA) is a polysaccharide of natural origin, which shows outstanding properties of biocompatibility, gel-forming ability, nontoxicity, and biodegradability [20,21]. The simple method of SA gelation using divalent cations, e.g., Ca^2+^, allows to form microspheres [22], bulk disks [23], and porous freeze-dried scaffolds [24,25] with physical and biochemical properties required for bone tissue repair.

Alginate can be also processed as a hydrogel scaffold material using the 3D printing technique [26,27], which, unlike traditional methods, allows for the efficient and precise fabrication of bone substitutes with a very complex configuration and high resolution, designed specifically for a particular patient [28].

Scaffolds can be printed by the computer-aided design/manufacture (CAD/CAM) system directly from the bioactive material, eliminating the stages of mold preparation and gene incorporation procedures. Three-dimensional-printed scaffolds, as a rule, have controlled pore size, pore interconnectivity, and overall porosity, crucial for gradual biomolecule delivery [29], cellular growth, and cell–cell interactions [30]. In particular, grid-like structures, usually formed during 3D printing [31], were shown to maintain cell viability while facilitating the diffusion of nutrients [32] and provide sufficient drug release while exhibiting a high surface area [33]. Moreover, hydrogel scaffolds with similar-to-native bone pore sizes of 300–500 µm promote active vascularization in vivo [34] and can offer optimal osteoconductive properties resulting in enhanced bone regeneration after implantation [35,36].

The existing 3D printing techniques require the increasing complexity of the experimental setup [37,38] or the addition of extra steps [26,39] for effective polymerization and the layer-by-layer formation of SA-based 3D structures. Furthermore, the most commonly used method of gene incorporation via adsorption [40,41] does not provide adequate transfection kinetics for tissue regeneration. We have recently demonstrated the development of the gene-activated SA-based scaffold (GAS) fabrication platform using our original 3D cryoprinting technique [42,43] and showed it to overcome the mentioned difficulties. The present study aims to further extend this approach to demonstrate for the first time the osteogenic properties of such hydrogel scaffolds with embedded BMP-2 plasmids both in vitro and in vivo.

## 2. Results and Discussion

### 2.1. 3D Printing of Alginate GASs

**Figure 1:**
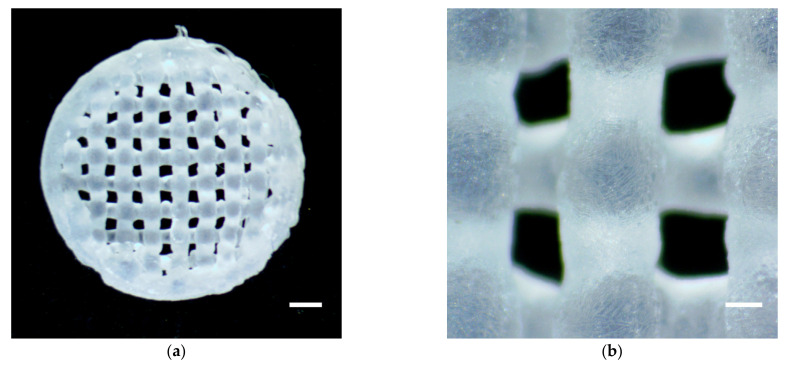
SA-based gene-activated hydrogel scaffold formed with 3D cryoprinter. (**a**) General view. Light microscopy. Scale bar 1 mm; (**b**) Microstructure. Light microscopy. Scale bar 200 µm.

Gene-activated hydrogel scaffolds were fabricated using the 3D cryoprinting technique. pDNA/PEI polyplexes were incorporated into the SA solution prior to the 3D printing process. The typical structure of the 3D cryoprinted gene-activated scaffold is shown in Figure 1. The formed, according to the 3D model, mesh-like hydrogel disk with a diameter of 8 mm and resolution of 500 µm meets the requirements for in vitro experiments and matches the critical-sized bone defect in rat parietal bone, formed for in vivo experiments, simultaneously.

### 2.2. Transfection Kinetics Study

**Figure 2:**
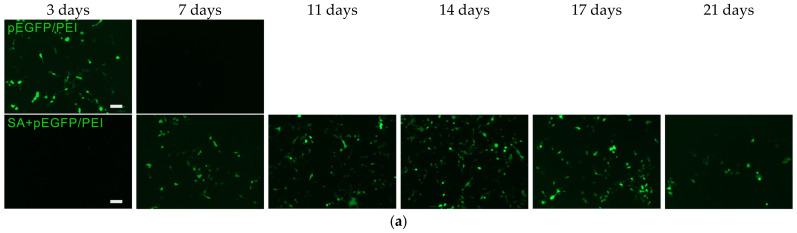

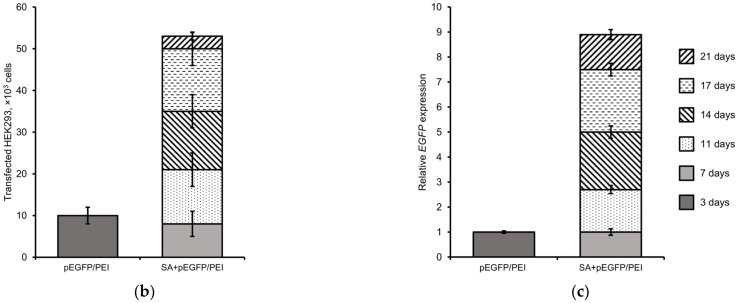
Transfection kinetics in vitro. (**a**) Transfected HEK293 after incubation with GASs impregnated with pEGFP/PEI. Fluorescent microscopy. Scale bar 100 µm; (**b**) The number of transfected HEK293 cells during their incubation with gene-activated alginate scaffolds. Flow cytometry; (**c**) Relative *EGFP* expression level during incubation of MSCs with GASs impregnated with pEGFP/PEI. Real-time PCR.

The transfection of HEK293 using GASs with pEGFP/PEI is shown in Figure 2. Fluorescence microscopy (Figure 2a) enables to qualitatively demonstrate the transfection kinetics of cells during their incubation with gene-activated scaffolds. Quantitative data are represented in Figure 2b,c. pEGFP/PEI polyplexes were shown to lose their transfecting ability after 7 days of incubation with HEK293, resulting in 10 ± 2% of transfected cells in 3 days. Whereas GASs facilitate the postponed (for 3 days), but prolonged (for 17 days), polyplex release that led to a stable transfection with an average rate of four thousand cells per day. The *EGFP* expression level in HEK293 correlated with the number of transfected cells calculated, confirming the efficacy of GAS application for gene delivery.

### 2.3. Gene Transfection In Vitro

**Figure 3:**
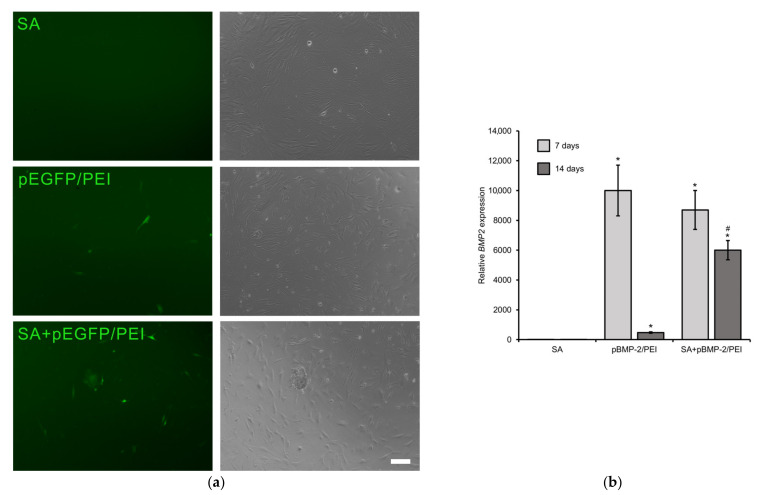
Transfection in vitro. (**a**) Transfected MSCs after 3 days of incubation with pEGFP/PEI and after 7 days of incubation with gene-activated scaffolds impregnated with pEGFP/PEI. Fluorescent (left) and light (right) microscopy. Scale bar 100 µm; (**b**) Relative *BMP2* expression level after 1 and 2 weeks incubation of MSCs with gene-activated scaffolds impregnated with pBMP-2/PEI. Real-time PCR. *—*p* < 0.05 (vs. SA) and #—*p* < 0.05 (vs. pBMP-2/PEI).

The transfecting ability of the polyplexes released from the GASs is shown in Figure 3. The model pEGFP was used to qualitatively show that when incubating with MCSs, the released pEGFP/PEI transfection capacity at day 7 was comparable to the positive control group (pBMP-2/PEI) at day 3 (Figure 3a) as predicted by the transfection kinetics study. Furthermore, when using GASs with therapeutic pBMP-2, the transfection of MSCs after 1 week of incubation led to a significant increase (almost 9000-fold) in *BMP2* gene expression levels (Figure 3b), which is of the same order as the gene level in the positive control group. Moreover, further incubation of MSCs with GASs showed the preservation of a gene expression level that was 12 times higher than in the positive control group.

The choice of the *BMP2* gene was dictated by the effect of bone morphogenetic protein 2 on MSC osteogenic differentiation [44,45]. However, the success of implementing a new bone substitute not only depends on the bioactive molecule, but also on the ability of the released from a scaffold DNA to transfect cells over time. The chosen method of incorporation of polyplexes comprising pEGFP and pBMP-2 plasmids into a sodium alginate solution, as well as their processing during 3D cryoprinting, had virtually no effect on their transfecting ability compared to the initial polyplexes. Moreover, the incorporation of polyplexes prior to the 3D printing enabled to form GASs that promote, compared to other studies [46,47], a postponed and more prolonged polyplex release resulting in the long-lasting transfection of cells. This is particularly crucial for the in vivo studies to avoid the acute inflammation phase after implantation and provide effective gene delivery.

### 2.4. Osteoinductive Properties of Gene-Activated Scaffolds In Vitro

The quantitative assessment of the BMP-2 abundance level in the culture supernatant at 1 and 2 weeks after MSC incubation with gene-activated scaffolds is shown in Table 1. The total BMP-2 concentration in the medium increased by 20 times when using GASs with pBMP-2/PEI relative to the negative control group, whereas there was only a 9-fold increase in the protein level when incubating only with pBMP-2/PEI.

**Figure 4:**
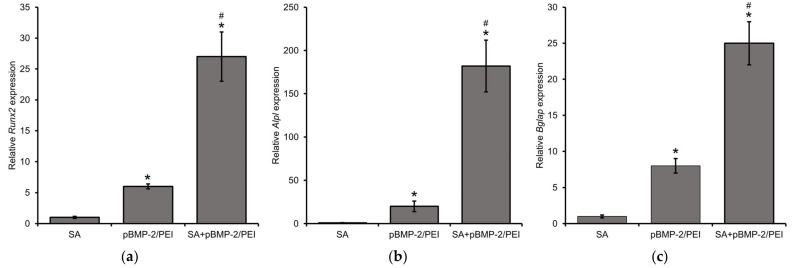
Relative gene expression levels of osteogenic markers: (**a**) *Runx2*, (**b**) *Alpl*, and (**c**) *Bglap* after 2 weeks incubation of MSCs with gene-activated scaffolds impregnated with pBMP-2/PEI. Real-time PCR. *—*p* < 0.05 (vs. SA) and #—*p* < 0.05 (vs. pBMP-2/PEI).

The amount of released BMP-2 is sufficient to induce osteogenic differentiation confirmed with the increase in the gene expression levels of the osteogenic markers *Runx2*, *Alpl,* and *Bglap*, which were determined after 2 weeks MSC incubation with GASs (Figure 4). *Runx2*, *Alpl,* and *Bglap* gene expression levels significantly increased by 27, 180, and 25 times, respectively. When incubating MSCs only with pBMP-2/PEI polyplexes, the *Runx2*, *Alpl,* and *Bglap* gene levels were 6, 20, and 8 times higher in comparison with the negative control group.

It is of great importance to evaluate cell osteodifferentiation in vitro in order to predict the possible efficacy of the developed gene-activated scaffold in vivo [48,49]. In our study, gene-activated SA-based hydrogel scaffolds, bearing pBMP-2/PEI polyplexes, were shown to promote MSC osteodifferentiation and were highly competitive with previously developed bioactive matrices [50]. The contradicted results of the *BMP2* gene expression level and protein synthesis after 1 week of incubation can be explained by the protein destruction due to its short half-life [51] in the positive control group and the preservation of BMP2 via adsorption [52] in the experimental group. Nevertheless, there was almost a 3-fold increase in the level of BMP-2 abundance in the culture supernatant after 2 weeks of MSC incubation with GASs compared to bare pBMP-2/PEI polyplexes. That resulted in the higher relative expression levels of the main osteogenic markers specific to both early (*Runx2* and *Alpl*) and late (*Bglap*) stages of osteogenesis, suggesting the perspective of an effective application of gene-activated scaffolds for prolonged gene delivery in vivo.

### 2.5. Osteoinductive Properties of Gene-Activated Scaffolds In Vivo

**Figure 5:**
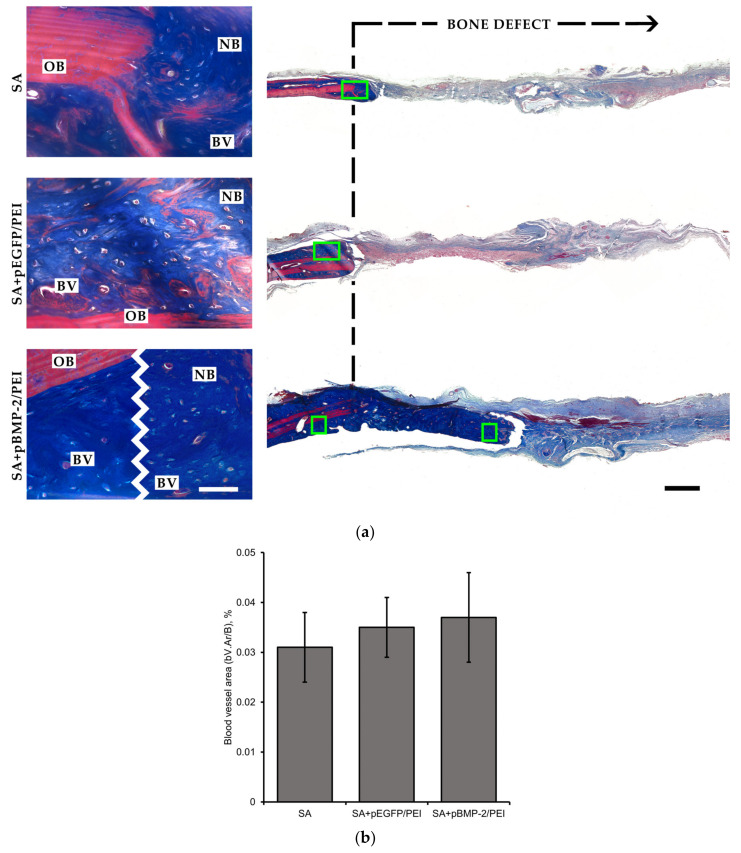
Bone regeneration in vivo. (**a**) Representative histologic sections at 8 weeks after implantation of SA scaffolds into a critical-sized cranial defect. New bone formation in the defects treated with scaffold only; GAS with pEGFP/PEI and GAS with pBMP-2/PEI. Masson’s trichrome staining. Light microscopy. OB—old bone. NB—new bone. BV—blood vessel. Scale bar 50 µm (**left**) and 500 µm (**right**). (**b**) Assessment of the blood vessel area in new bone tissue.

At 8 weeks, Masson’s trichrome staining of the obtained sections revealed the old (matured) and the new (woven) bone areas, as well as fibrous connective tissue within the defect (Figure 5a). A substantial difference in the bone regeneration degree was observed between the control group and GASs with pBMP-2/PEI polyplexes. Blank SA scaffold when implanted was surrounded by thin connective tissue with a negligible amount of new bone tissue, whereas the implantation of the SA scaffold containing pBMP-2/PEI resulted in fibrous tissue and conspicuous new bone formation in the defect zone. Moreover, the bone structure in both groups was characterized by a high osteoid density and active vascularization. There was no statistical difference in blood vessel volume among the groups (Figure 5b). This may imply no influence of genetic constructs on the new bone morphology. The implantation of scaffolds with pEGFP/PEI polyplexes showed the same bone defect filling as the control group meaning that the efficient bone regeneration was stimulated with pBMP-2 specifically.

**Figure 6:**
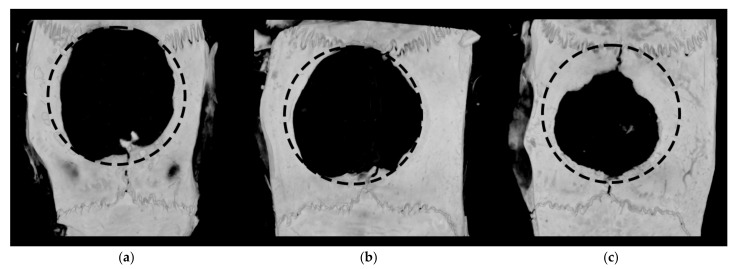
Representative 3D reconstruction images at 8 weeks after SA scaffold implantation into a critical-sized cranial defect. New bone formation in the defects treated with (**a**) scaffold only; (**b**) GAS with pEGFP/PEI; and (**c**) GAS with pBMP-2/PEI. Micro-CT. Dashed line—initial defect zone with a diameter of 7.5 mm.

Radiographic images of bone defects captured at 8 weeks after scaffold implantation are presented in Figure 6. The defects treated with blank SA scaffolds, as well as scaffolds containing pEGFP/PEI polyplexes, maintained their circular shape and showed minimal new bone formation from the periphery. On the contrary, defects treated with scaffolds containing pBMP-2/PEI were filled with the new bone that expanded further to the defect center demonstrating effective osteoinductive properties of developed GASs with the pBMP-2/PEI polyplexes.

The results of the histomorphometric and micro-CT analyses are summarized in Table 2. According to the histomorphometric analysis at 8 weeks after scaffold implantation, the amount of bone tissue to the amount of full tissue (B/T ratio) within the defect zone was 5 ± 2%, 3 ± 2%, and 25 ± 7% for blank scaffolds, scaffolds with pEGFP/PEI, and scaffolds with pBMP-2/PEI, respectively. According to the micro-CT analysis at 8 weeks, after scaffold transplantation, the new bone volume (Nb. V) within the defect zone was 10 ± 3%, 11 ± 4%, and 46 ± 11% for blank scaffolds, scaffolds with pEGFP/PEI, and scaffolds with pBMP-2/PEI, respectively.

The development of optimal technology to form highly effective gene-activated scaffolds is still relevant and challenging in biomaterial science and tissue engineering [11]. The osteoinduction in vivo seems to be the most crucial criterion to evaluate when developing GASs for bone regeneration, though it can vary in different studies. Thus, the recent research demonstrated that the implantation of hydrogel scaffolds with ex situ-transfected stem cells did not differ from in situ gene delivery [53], resulting in insufficient bone regeneration. In [54], the comparison of the ex situ and in situ gene delivery was also introduced, and not only was the in vivo bone formation more significantly improved in situ than ex vivo, but the nonviral system efficiency was also similar to the viral one.

In our study, the *BMP2* gene was introduced into host cells using scaffold-based in situ transfection, avoiding complicated processes related to cells. It should also be highlighted that 3D printing technology ensured the simplification of the GAS fabrication process, eliminating the loss of DNA during its incorporation into the scaffold, and provided a high integration of porous scaffold with the tissue in the implantation zone. That resulted in the more distinct osteoinduction properties of the 3D printed SA-based scaffolds compared to the traditional hydrogel scaffolds [55] and even to the artificially modified (physically and chemically) matrices [56,57]. Thus, the developed platform of the gene-activated scaffold formation for bone regeneration is a promising methodology that is expected to be widely applied in bone defect restoration and bone disease treatment.

## 3. Conclusions

The present study is aimed at a comparative assessment of neo-osteogenesis in critical-sized bone defects using SA-based hydrogel scaffolds fabricated by 3D cryoprinting. We have previously demonstrated that 3D cryoprinted gene-activated scaffolds maintain cell viability and provide a prolonged plasmid release in a high concentration sufficient for the effective transfection of cells in vitro and in vivo [43]. The focus of this study is on implementing the developed universal platform to form GASs in which the model plasmids were replaced with therapeutic ones for efficient bone regeneration enhancement.

The results of our studies, in our opinion, quite convincingly show the possibility of the effective use of three-dimensional constructs from gene-activated biopolymers to replace bone defects of critical sizes. Modern approaches to the design and fabrication of personalized osteoinductive and osteoconductive tissue engineering structures based on such materials using advanced additive manufacturing technologies that are currently being developed by us, as well as by many other researchers, may, in the very near future allow solving such problems directly in real clinics.

## 4. Materials and Methods

### 4.1. Plasmid DNA

Plasmid DNA-encoding enhanced green fluorescent protein (pEGFP, Clontech, Mountain View, CA, USA) and human bone morphogenetic protein-2 (pBMP-2, Eurogen, Moscow, Russia) were used as primary genetic constructs. Plasmids were amplified in Escherichia coli in LB broth medium (Merck KGaA, Darmstadt, Germany) with 50 µg/ mL of kanamycin (GRISP, Porto, Portugal) and isolated with a Zymo Research Plasmid Midiprep Kit (Zymo Research, Irvine, CA, USA) according to the manufacturer’s protocol.

Polyethyleneimine (PEI, linear, 25 kDa, Polysciences, Warrington, PA, USA) was used as a transfecting agent. To form polyplexes, pDNA and PEI were mixed in a 1:3 ratio in 100 µL of diH_2_O at 37 °C for 30 min.

### 4.2. GAS Formation

Gene-activated sodium alginate scaffolds were fabricated using 3D cryoprinting as described earlier [43]. Briefly, 8 mg of SA was dissolved in 92 µL of distillated H_2_O, containing 20 µg pDNA/60 µg PEI for in vitro study and 100 µg pDNA/300 µg PEI for in vivo study. The polymer composition was then loaded into the bespoke 3D cryoprinter [42] and dispensed to a platform at −10 °C according to the 3D computer model (disk with a diameter of 8 mm and a thickness of 3 mm with a mesh size of 1 × 1 mm^2^). Subsequent polymerization was carried out in 10 wt. % chloride calcium (Merck KGaA, Darmstadt, Germany) aqueous solution at 20 °C for 1 h. The scaffolds were then dried and stored at 4 °C before being applied in in vitro and in vivo studies. All stages were carried out under sterile conditions.

### 4.3. Cell Culture

To study polyplex transfection kinetics, human embryonic kidney 293 (HEK293) cells were used. To investigate the transfecting ability of the released polyplexes and the GAS influence on cell osteodifferentiation mesenchymal stem cells (MSCs), derived from rat adipose tissue, 3–4 passages were used [58]. Cells were incubated in growth medium: DMEM/F12 (PanEco, Moscow, Russia), 10% fetal bovine serum (FBS, PAA Laboratories, Etobicoke, ON, Canada), 0.584 mg/mL L-glutamine (PanEco, Moscow, Russia), 5000 u/mL streptomycin (PanEco, Moscow, Russia), and 5000 u/mL penicillin (PanEco, Moscow, Russia) in Petri dishes under standard culture conditions (37 °C, 5% CO_2_).

### 4.4. Transfection Kinetics

HEK293 cells were detached from the surface of Petri dishes using a Versene solution with 0.25% trypsin and seeded into 24-well plates with a density of 1 × 10^6^ cells per well. GASs with pEGFP/PEI were added to the wells, and transfecting ability of the released pEGFP/PEI was assessed every 3–4 days for 21 days of incubation with GASs.

The transfection process was studied using fluorescence microscopy on a Zeiss Axio Observer.D1 microscope (Carl Zeiss Microscopy GmbH, Oberkochen, Germany). To determine the number of transfected cells, they were removed from the surface of the wells and centrifuged at 1200 rpm for 5 min, and the number of cells synthesizing EGFP was counted on a flow cytometer CyFlow^®^ Space (Partec, Canterbury, UK). The analysis was carried out using FloMax software. The expression level of the *EGFP* gene was analyzed by real-time PCR using the intercalating dye “SYBR Green I” (Eurogen, Moscow, Russia). Total RNA was isolated from cells using the RNeasy Plus Mini Kit (Qiagen, Hilden, Germany). cDNA synthesis was carried out using the RevertAid kit (Thermo Fisher Scientific, Waltham, MA, USA). The expression level of the analyzed gene was normalized by the expression values of the reference genes: *GAPDH* and *ACTB*.

Every 3–4 days, the scaffold was transferred to a new 24-well plate, and the procedure was repeated. The wells with 2 µg pDNA/6 µg PEI were used as a positive control group to evaluate the transfection at 7 days, and formed polyplexes were incubated in cultural medium under 37 °C and 5% CO_2_ for 3 days and were added to the wells afterward.

### 4.5. Transfection Efficacy In Vitro

MSCs were detached from the surface of Petri dishes using a Versene solution with 0.25% trypsin and seeded into 24-well plates with a density of 1 × 10^5^ cells per well. GASs with pEGFP/PEI or pBMP-2/PEI were added to the wells, and transfecting ability of the released pDNA/PEI was assessed 7 and 14 days after incubation with pEGFP and pBMP-2, respectively. Cells transfected with pEGFP/PEI were observed with fluorescent microscopy using Axio Observer (Carl Zeiss, Oberkochen, Germany). The released pBMP-2/PEI transfection efficacy was evaluated by real-time PCR. The wells without plasmid DNA (scaffolds only) were used as a negative control group and with 2 µg pDNA/6 µg PEI—as a positive control group. To evaluate the transfection in 14 days, polyplexes formed as a positive control group were incubated in cultural medium under 37 °C and 5% CO_2_ for 7 days and were added to the wells afterwards.

### 4.6. Osteodifferentiation In Vitro

For osteodifferentiation assay, MSCs were seeded in 24-well plates with a density of 2.5 × 10^4^ cells per well and incubated in osteogenic medium: DMEM (PanEco, Moscow, Russia), 10% fetal bovine serum (FBS, PAA Laboratories, Etobicoke, ON, Canada), 0.584 mg/ mL L-glutamine (PanEco, Moscow, Russia), 5000 u/mL streptomycin (PanEco, Moscow, Russia), 5000 u/mL penicillin (PanEco, Moscow, Russia), 10 mM β-glycerol phosphate (Merck KGaA, Darmstadt, Germany), and 50 µg/mL ascorbic acid (Merck KGaA, Darmstadt, Germany). Cells were incubated with blank SA scaffolds (negative control group), with GASs (experimental group), and with 2 µg pDNA/6 µg PEI (positive control group) for 14 days. Half of the medium was replaced twice a week.

RNA was isolated from cell cultures using the RNeasy Plus Mini Kit (Qiagen, Hilden, Germany) and reverse-transcribed into cDNA using the RevertAid Kit (Thermo Fisher Scientific, Waltham, MA, USA). Expression levels of osteogenic differentiation markers *Runx2*, *Alpl,* and *Bglap* were evaluated by real-time PCR using SYBR Green intercalating dye (Eurogen, Moscow, Russia). Reactions were carried out at 95 °C for 6 min, 40 cycles of 95 °C for 10 s, gradient 59.6/64.6 °C for 30 s, and 72 °C for 20 s, followed by melting curve analysis. *Gapdh* and *Actb* were used as endogenous reference genes. The analyzed gene expression levels were normalized by the expression values of the negative control group.

For BMP-2 identification and quantitation, half of the culture of the supernatant was collected every 3 days and condensed using Millipore Amicon 3 kDa spin column (Merck KGaA, Darmstadt, Germany) after 7 and 14 days of incubation. The secretion levels of BMP-2 were analyzed by an enzyme-linked immunosorbent assay (ELISA) kit (R&D Systems, Minneapolis, MN, USA) according to the manufacturer’s protocol.

### 4.7. In Vivo Study

**Figure 7:**
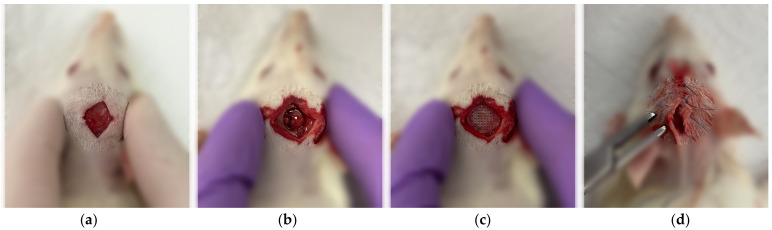
The surgical procedure of the scaffold implantation into the critical-sized cranial defect in rats. (**a**) Skin incision; (**b**) Trephine defect in parietal bone; (**c**) Scaffold positioning; and (**d**) Skin suturing.

Bone regeneration in vivo was studied on a model of a critical-sized cranial defect in Wistar rats weighing 200 g (n = 9) (Figure 7). Rats were anesthetized (Zoletil 30 mg/kg and Xylazine 5 mg/kg), and the top of the head was shaved and disinfected with ethanol. Then, a skin incision was made in the center above the sagittal suture, and a trephine defect 7.5 mm in diameter was created in the parietal bone under saline solution irrigation. The scaffolds placed in the defect zone were divided into 3 groups: blank scaffolds (SA), scaffolds with model pEGFP/PEI polyplexes (SA + pEGFP/PEI), and scaffolds with pBMP-2/PEI polyplexes (SA + pBMP-2/PEI). The defects were then closed with sutures. To prevent infectious complications, 10 µg/kg of Ceftriaxone (Biochemist, Saransk, Russia) was intramuscularly injected.

Eight weeks after surgery, rats were euthanized by CO_2_ inhalation, and the cranial bones containing the transplanted sites were removed. These bone blocks were then fixed with 10% formaldehyde for 2 days and studied using micro-CT and histological examinations with morphometric analysis.

All experiments were approved by the local bioethical committee of Sechenov University (№ PRC-079 from 6 April 2021) in compliance with the Guide for the Care and Use of Laboratory Animals published by the US National Institutes of Health (NIH publication no. 85–23, revised 1996), European Convention for the Protection of Vertebrate Animals used for Experimental and Other Scientific Purposes, and ISO 10993–22006.

### 4.8. Micro-CT

The samples were scanned using a high-resolution micro-CT (Skyscan 1276, Bruker, Kontich, Belgium) with an X-ray voltage of 60 kV, aluminum filter 0.5 mm, and isotropic voxel size 10 µm. After standardized reconstruction using NRecon software, 3D images were analyzed using Dragonfly software (v2021.3, ORS, Montreal, QC, Canada). For quantitative evaluation of the newly formed bone, the new bone volume (Nb. V) was calculated relative to the initial defect volume.

### 4.9. Histology

After micro-CT, the samples were decalcified in EDTA for 3 weeks, then dehydrated in ascending alcohol and embedded in paraffin. To assess the bone regeneration degree, the sections closest to the center of each defect were obtained and stained with Masson’s trichrome (Biovitrum, Saint-Petersburg, Russia) according to the manufacturer’s protocol. Images were captured using light microscopy (Zeiss Axio Observer.D1, Carl Zeiss Microscopy GmbH, Oberkochen, Germany), and evaluation of the bone area/tissue area (B/T) ratio and the blood vessel area/bone area (bV.Ar/B) ratio within the defect was carried out.

### 4.10. Statistical Analysis

All data were presented as μ ± SD. No less than 6 experimental replicates were studied. Statistical analysis and graphing were performed with SigmaPlot v14.0 (Systat Software Inc., Palo Alto, Santa Clara, CA, USA). The differences among groups were assessed by one-way ANOVA using Tukey’s post hoc tests. Statistical significance was accepted for *p* < 0.05.

## Figures and Tables

**Table 1:** BMP-2 abundance level (pg/mL) in the culture supernatant when incubating MSCs with gene-activated scaffolds for 1 and 2 weeks. ELISA.

	SA	pBMP-2/PEI	SA + pBMP-2/PEI
1 week	105 ± 7	440 ± 30 *	760 ± 30 *^,#^
2 weeks	110 ± 5	960 ± 70 *	2150 ± 110 *^,#^

* *p* < 0.05 (vs. SA) and # *p* < 0.05 (vs. pBMP-2/PEI).

**Table 2:** Comparative assessment of the new bone formation degree using histomorphometric and micro-CT analyses at 8 weeks after SA scaffold implantation.

	SA	SA + pGFP/PEI	SA + pBMP-2/PEI
Histomorphometric Analysis (B/T, %)	5 ± 2	3 ± 2	25 ± 7 *
Micro-CT Analysis (Nb. V, %)	10 ± 3	11 ± 4	46 ± 11 *

* *p* < 0.05 (vs. SA and SA + pGFP/PEI).

## Data Availability

Available upon request.
